# Manual therapy with and without vestibular rehabilitation for cervicogenic dizziness: a systematic review

**DOI:** 10.1186/2045-709X-19-21

**Published:** 2011-09-18

**Authors:** Reidar P Lystad, Gregory Bell, Martin Bonnevie-Svendsen, Catherine V Carter

**Affiliations:** 1 Department of Chiropractic, Macquarie University, Sydney, Australia

**Keywords:** Cervicogenic dizziness, Vertigo, Manual therapy, Vestibular rehabilitation, Spinal manipulation, mobilisation

## Abstract

**Background:**

Manual therapy is an intervention commonly advocated in the management of dizziness of a suspected cervical origin. Vestibular rehabilitation exercises have been shown to be effective in the treatment of unilateral peripheral vestibular disorders, and have also been suggested in the literature as an adjunct in the treatment of cervicogenic dizziness. The purpose of this systematic review is to evaluate the evidence for manual therapy, in conjunction with or without vestibular rehabilitation, in the management of cervicogenic dizziness.

**Methods:**

A comprehensive search was conducted in the databases Scopus, Mantis, CINHAL and the Cochrane Library for terms related to manual therapy, vestibular rehabilitation and cervicogenic dizziness. Included studies were assessed using the Maastricht-Amsterdam criteria.

**Results:**

A total of fifteen articles reporting findings from thirteen unique investigations, including five randomised controlled trials and eight prospective, non-controlled cohort studies were included in this review. The methodological quality of the included studies was generally poor to moderate. All but one study reported improvement in dizziness following either unimodal or multimodal manual therapy interventions. Some studies reported improvements in postural stability, joint positioning, range of motion, muscle tenderness, neck pain and vertebrobasilar artery blood flow velocity.

**Discussion:**

Although it has been argued that manual therapy combined with vestibular rehabilitation may be superior in the treatment of cervicogenic dizziness, there are currently no observational and experimental studies demonstrating such effects. A rationale for combining manual therapy and vestibular rehabilitation in the management of cervicogenic dizziness is presented.

**Conclusion:**

There is moderate evidence to support the use of manual therapy, in particular spinal mobilisation and manipulation, for cervicogenic dizziness. The evidence for combining manual therapy and vestibular rehabilitation in the management of cervicogenic dizziness is lacking. Further research to elucidate potential synergistic effects of manual therapy and vestibular rehabilitation is strongly recommended.

## Background

Dizziness is a non-specific symptom that is commonly encountered by primary health care practitioners [1], and the prevalence has been reported to be between 11.1% and 28.9% [2-5]. It can be experienced as faintness, unsteadiness, perception of spinning and disorientation [6-8]. The mechanisms producing these symptoms are multiple and can involve several different organ systems. Ardc, Topuz and Kara [9] reported the most frequent diagnosis of patients suffering from dizziness to be benign paroxysmal positional vertigo, endolymphatic hydrops, migraine, central decompensation, acute vestibulopathy and autonomic dysfunction. Furthermore, it is not uncommon for patients experiencing dizziness to have more than one diagnosis. Dizziness is commonly seen in whiplash patients, affecting 20-58% of individuals with flexion-extension injuries [10].

One specific type of dizziness is cervicogenic dizziness. The disorder was first described as "cervical vertigo" by Ryan and Cope in 1955 [11]. Other terms used for the same disorder are proprioceptive vertigo, cervicogenic vertigo and cervical dizziness [12-14]. Although the diagnosis has remained controversial since its introduction, several observations have led to the proposal of a plausible pathophysiological mechanism. The deep intervertebral muscles in the cervical spine possess a high density of muscle spindles and are assumed to play an important role in postural control [15-18]. Cervical afferents are known to be involved in the cervico-collic reflex, the cervico-ocular reflex and the tonic neck reflex, which work in conjunction with other reflexes influenced by visual and vestibular systems to stabilise the head, the eyes and posture [19]. Vestibular and proprioceptive input is linearly combined for computing egocentric, body-centred coordinates [20].

Several authors have demonstrated that anaesthetic injections to the upper cervical dorsal nerve roots can produce dizziness and nystagmus [21-23]. Electrical stimulation to cervical muscles has also been shown to induce a sensation of tilting or falling [24]. Brandt and Bronstein [25] proposed a mechanism where changed firing characteristics of cervical somatosensory receptors due to neck pain lead to a sensory mismatch between vestibular and cervical input, resulting in cervical vertigo.

Several authors have proposed manual therapy interventions for the treatment of dizziness of a cervical origin [26-28]. Indeed, it has been suggested that the management of cervicogenic dizziness should be the same as for cervical pain [25]. In a systematic review of the literature, Reid and Rivett [29] concluded that there is limited evidence to support manual therapy treatment of cervicogenic dizziness. Moreover, it was recommended that further research be conducted, especially randomised controlled trials (RCTs), to provide more conclusive evidence of the role of manual therapy for cervicogenic dizziness.

Another treatment modality that is advocated for cervical pain is sensorimotor rehabilitation exercises [19,30]. These exercises fall under the scope of exercises included in vestibular rehabilitation therapy. Vestibular rehabilitation emerged as a group of exercises for peripheral vestibular disorders, aiming to maximise central nervous system compensation to vestibular pathology [31,32]. These exercises are usually movement based, and can be further subcategorised according to different physiological rationales: (i) compensatory responses using motion to habituate activity in the vestibular nuclei; (ii) adaptation for visual-vestibular interaction and possibly eye/hand coordination, using repetitive and provocative movements of the head and/or eyes; (iii) substitution which promotes the use of individual or combinations of sensory inputs to bias use away from dysfunctional vestibular input; (iv) postural control exercises, falls prevention, relaxation training, reconditioning activities and functional/occupational retraining, which are based on motor learning principles [33,34].

Hillier and Hollohan [34] concluded that there was moderate to strong evidence that vestibular rehabilitation is safe and effective in the management of unilateral peripheral vestibular disorders. Moreover, several authors encourage the implementation of vestibular rehabilitation in treatment of dizziness of a cervical origin [10,32,35], and published case studies have reported positive outcomes when combining manual therapy and vestibular rehabilitation [36,37].

To the authors' knowledge, the evidence of implementing vestibular rehabilitation with manual therapy in the management of cervicogenic dizziness has not been systematically reviewed. Thus, the purpose of this systematic review was: (i) to provide an updated systematic review of manual therapy for cervicogenic dizziness by including higher level evidence published since the previous review by Reid and Rivett [29], and (ii) to compare the evidence of (a) manual therapy with vestibular rehabilitation for cervicogenic dizziness with (b) manual therapy without vestibular rehabilitation for cervicogenic dizziness.

## Methods

This systematic review adhered to the guidelines outlined in the Preferred Reporting Items for Systematic Reviews and Meta-Analyses (PRISMA) Statement [38].

### Eligibility criteria

This systematic review was limited to prospective, controlled or non-controlled intervention studies published in peer-reviewed journals. Retrospective study designs, case reports, case series, commentaries, letters to the editor, and expert opinions were excluded from this review. No language restrictions were applied in this review.

Eligible studies had to investigate a cohort of patients diagnosed with cervicogenic dizziness. Cervicogenic dizziness was defined as the presence of dizziness, imbalance or unsteadiness related to movements or position of the cervical spine, or occurring with a stiff or painful neck [29]. Studies investigating populations diagnosed with cardiovascular disorders, central nervous system disorder (e.g. cerebellar ataxia, stroke, demyelination), Mal de Debarquement syndrome, migraine-associated vertigo, psychogenic dizziness, vestibular disorders (e.g. benign paroxysmal positional vertigo, Meniere's disease, peripheral vestibulopathy), were not included in this review. Studies were also excluded if the study population was comprised of patients with a history of active inflammatory joint disease, spinal cord pathology, cervical spine cancer or infection, bony disease or marked osteoporosis, marked cervical spine disc protrusion, acute cervical nerve root symptoms, fracture or dislocation of the neck, or previous surgery to the upper cervical spine.

This review considered two possible interventions, namely manual therapy alone and manual therapy in conjunction with vestibular rehabilitation. For the purposes of this review, manual therapy was defined as spinal manipulation (high velocity, low amplitude techniques) or mobilisation (low-velocity, small or large amplitude techniques) [29]. Vestibular rehabilitation was defined as an exercise-based group of approaches with the aim of maximising the central nervous system compensation for vestibular pathology [39]. Vestibular rehabilitation techniques included habituation (movement provoking) with gaze stabilising (adaptation), sensory substitution, and balance and gait/activity training [34].

### Search Strategy

A comprehensive search of the literature was conducted, including electronic searches of the Scopus, Mantis, and CINAHL databases from January 1955 to June 2010. In addition, the Cochrane Library was searched from inception (1993) to June 2010 to identify any relevant Cochrane Reviews. Keywords used in the literature search included "cervicogenic dizziness" and "manual therapy". Alternative spellings, synonyms and related terms, and truncated versions of both the condition and the intervention were included. In addition, bibliographies of included studies and relevant review articles were hand searched to indentify potentially eligible studies not captured by the electronic searches.

### Study selection

Citations from the electronic searches were combined in a single list and duplicate records were discarded. Two reviewers screened all titles and abstracts to identify and remove obviously irrelevant citations. Full text versions of all potentially eligible articles were retrieved and evaluated by two independent reviewers to determine eligibility for inclusion in this review. Any differences were resolved by mutual consensus with a third independent reviewer.

### Data extraction process

Data from eligible studies were extracted and compiled in a spreadsheet. For the purposes of this systematic review the following data were extracted: (i) study population (e.g. age, gender, diagnosis, and sample size); (ii) study design; (iii) intervention; (iv) outcome measures; and (v) main findings.

### Data analysis

Owing to the clinically heterogeneous nature of the included studies (i.e. varying study designs, interventions, outcome measures, and quality of data), a meta-analysis was deemed unfeasible. Thus, in this review only a qualitative analysis of included studies was undertaken. As per the previous review by Reid and Rivett [29], qualitative analysis was achieved by attributing levels that rate the scientific evidence, i.e. Level 1: Strong evidence (provided by generally consistent findings in multiple higher quality RCTs); Level 2: Moderate evidence (provided by generally consistent findings in one higher quality RCT and one or more lower quality RCTs); Level 3: Limited evidence (provided by generally consistent findings in one or more lower quality RCTs); and Level 4: No evidence (if there were no RCTs or if the results were conflicting).

### Assessment of methodological quality

The methodological quality of the included studies was assessed using the Maastricht-Amsterdam criteria [40]. The Maastricht-Amsterdam criteria list, which consists of 19 items assessing patient selection, interventions, outcome measures and statistics, is included in Additional file 1. Two independent reviewers assessed methodological quality and any differences were resolved by mutual consensus with a third independent reviewer. Each item was answered "yes", "no", or "don't know", and one point was assigned for each "yes" (fulfilled item). The assessed studies were categorised as either poor, moderate or good based on the percentage of fulfilled items from the Maastricht-Amsterdam criteria list. In accordance with other authors using similar quality assessment methods, the cut-off percentage values were arbitrarily set at < 50% (poor), 50-80% (moderate), and > 80% (good) [41-43].

## Results

**Figure 1:**
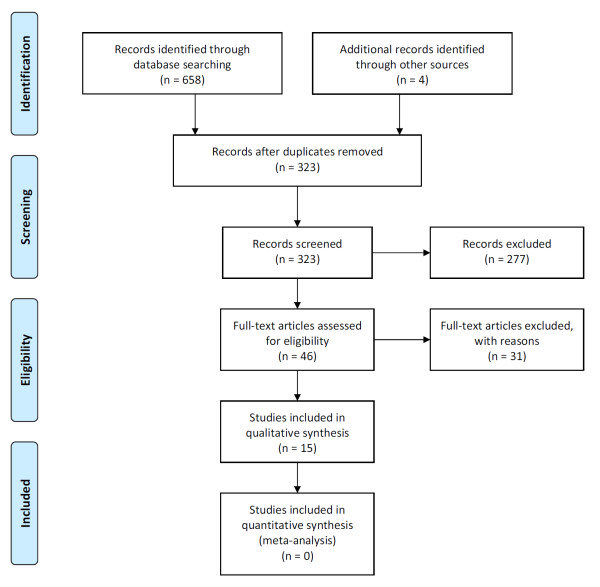
**PRISMA flow diagram**.

The electronic searches returned 658 hits, which included 335 duplicate records and 323 unique citations. After removing duplicate records and screening titles and abstracts to discard obviously irrelevant citations, a total of 42 potentially eligible studies were identified. A hand search revealed four additional studies that were not captured by the electronic search. Thus, a total of 46 potentially eligible studies were evaluated for inclusion in this systematic review. Thirty-one studies [29,36,44-72] did not meet the inclusion criteria and were excluded from this review. See Additional file 2 for a list of excluded studies including reasons for exclusion. Figure 1 contains a flow diagram of the study selection process. Two articles [26,73] reported data from the same RCT, and the results from one cohort study were published in two separate articles [74,75] Thus, this review included reports from a total of thirteen unique investigations. See Table 1 for a description of included studies.

**Table 1:** Included studies

Study	Participants	Interventions		Outcome measures	Main findings	Quality
					
		Manual therapy	Vestibular rehabilitation			
*Randomised controlled trials:*						
Karlberg et al. 1996 [26]; Malmström et al. 2007 [44]	n = 34 (88% females) Mean age: 37 Age range: 25-55 Country: Sweden Setting: primary care centers and a tertiary referral center	Mobilisation; Soft tissue therapy (relaxation techniques, stabilisation techniques); Home training program; Ergonomic changes at work	Nil	Dizziness frequency; Dizziness intensity; Posturography	- Significantly improved dizziness intensity and neck pain post-treatment (p < 0.05). - Significantly improved postural sway post-treatment (p < 0.2). - 14 patients (82%) reported improvements at 6 months post-treatment.^†^ - 11 patients (65%) reported improvements in dizziness at 2 year post-treatment.^†^	Moderate

Reid et al. 2008 [47]	n = 34 (62% females), 1 drop-out Mean age: 63.5 Age range: not reported Country: Australia Setting: University of Newcastle	Mobilisation (SNAGs)	Nil	DHI; Dizziness severity (VAS); Dizziness frequency; Neck pain (VAS); Posturography	- Significantly reduced DHI, dizziness severity, dizziness frequency and neck pain in the treatment group at 6 and 12 weeks post-treatment (p < 0.05). - No difference in dizziness severity at 12 weeks post-treatment. - No difference in dizziness frequency at either 6 or 12 weeks post-treatment.	Good

Kang, Wang and Ye 2008 [48]	n = 76 (49% females) Mean age: 32.4 Age range: 18-45 Country: China Setting: hospital	*Group A:* Spinal manipulation *Group B:* Acupressure	Nil	TCD-US; TCM syndrome diagnostic criteria	- Significantly reduced VBA blood flow velocity post-treatment in both groups (p < 0.01). - Significantly larger reduction in left and right vertebral artery blood flow velocity in Group B compared with Group A (p < 0.01). - Group differences remained statistically significant at a 6-month follow-up.	Moderate

Fang 2010 [49]	n = 168 (73% females) Mean age: 37.5 Age range: not reported Country: China Setting: hospital	*Treatment group:* Spinal manipulation; Soft tissue therapy *Control group:* TCM medication	Nil	CVSFAS; Colour Doppler ultrasonography	- Significant improvements in dizziness (p < 0.01), shoulder/neck pain (p < 0.05), and headache (p < 0.01) post-treatment. - Significant reduction of cervical artery spasm index and atlantoaxial displacement index in the treatment group post-treatment (p < 0.05).	Moderate

Du et al. 2010 [50]	n = 70 (54% females) Mean age: 37.6 Age range: 21-45 Country: China Setting: hospital	*Treatment group:* Spinal manipulation; Soft tissue therapy *Control group:* Traction; Medication	Nil	CVSFAS; Radiography; TCD-US; TCM syndrome diagnostic criteria	- Significant improvements in dizziness scores, vertebral displacement post-treatment (p < 0.01). - Significantly reduced left and right vertebral artery blood flow velocity post-treatment (p < 0.01). - Significantly improved clinical outcomes six months post-treatment (p < 0.01).	Moderate

*Prospective cohort studies:*						

Konrad and Gerencser 1990 [51]	n = 54 (74% females) Mean age: 34.7 Age range: not reported Country: Hungary Setting: hospital	Mobilisation; Manipulation	Nil	Dizziness (instrument not specifically stated); Electronystagmography	- 40 patients (74%) experienced improvement of dizziness post-treatment.^†^	Poor

Mahlstedt, Westhofen and König 1992 [52]	n = 28 (gender distribution not reported) Mean age: not reported Age range not reported Country: Germany Setting: not reported	Spinal manipulation	Nil	No information provided	- 19 patients (68%) reported reduced dizziness post-treatment.^†^	Poor

Uhlemann et al. 1993 [53]	n = 12* (gender distribution not reported) Mean age: 40.7 Age range: not reported Country: Germany Setting: not reported	Mobilisation; Spinal manipulation (traction)	Nil	Cervical turn test	- 5 out of 9 patients went from testing positive to testing negative on the cervical turn test post-treatment.	Poor

Bracher et al. 2000 [54]	n = 15 (80% females), 3 drop-outs Mean age: 41 Age range: 27-82 Country: Brazil Setting: chiropractic clinic	Spinal manipulation; Soft tissue therapy; Electrotherapy; Labyrinth sedation medication; sEMG biofeedback; Exercise program	Nil	Dizziness (instrument not specifically stated; "improvement of symptoms was based on patient's reports")	- 9 patients (60%) reported complete remission of dizziness, 3 patients (20%) reported consistent improvement with rare recurrence of episodes of mild intensity, and 3 patients (20%) reported no change.^†^	Poor

Hülse and Hölzl 2000 [55]	n = 67 (52% females) Mean age: 49 Age range: 18-66 Country: Germany Setting: not reported	Soft tissue therapy (traction massage, PIR, occipital-base-release technique, atlas-impulse-therapy)	Nil	Craniocorpography; Posturography	- Significant improvements in pathological vestibulospinal reactions found post-treatment (p < 0.001).	Poor

Chen and Zhan 2003 [56]	n = 16 (38% females) Mean age: 42.4 Age range: 38-58 Country: China Setting: hospital	Spinal manipulation; Soft tissue therapy	Nil	TCD-US; Radiography; TCM syndrome diagnostic criteria	- 14 patients (87.5%) reported marked improvement or complete remission of symptoms. - Significantly decreased vertebral artery mean blood flow velocity post-treatment (p < 0.05). - Significantly reduced vertebral displacement post-treatment (p < 0.05).	Poor

Wu et al. 2006 [45]; Wu et al. 2008 [46]	n = 121 (73% females) Mean age: not reported Age range: 20-71 Country: China Setting: hospital	Tuina manipulation therapy (pressing-kneading manipulation applied continuously to bilateral vertebrae for 5 minutes)	Nil	Custom-made instrument to measure skull 3D motion and head repositioning.	- Significant improvements in skull spatial offset repositioning ability post-manipulation (p < 0.01).	Poor

Strunk and Hawk 2009 [57]	n = 21 (63% females), 2 drop-outs Mean age: 70 Age range: 44-85 Country: USA (California) Setting: Cleveland Chiropractic College	Spinal manipulation; Soft tissue therapy (myofascial release, PIR, and heat or cold therapy)	Nil	DHI SF-BBS NDI	- Improved DHI and SF-BBS scores.^†^ - Improved balance.^†^ - Decreased dizziness and neck pain.^†^	Moderate

CVSFAS: cervical vertigo, symptoms and functional assessment scale; DHI: Dizziness Handicap Inventory; NDI: Neck Disability Index; HVLA: high-velocity, low amplitude; PIR: post-isometric relaxation; RCT: randomised, controlled trial; ROM: range of motion; SF-SSB: Berg Balance Scale (short form); sEMG: surface electromyography; SNAGs: sustained natural apophyseal glides; TCD: transcranial Doppler ultrasonography; TCM: traditional Chinese medicine; VAS: Visual Analogue Scale.

* Of the 42 patients that were recruited for this study only 12 patients were included in the manual therapy group, of which only 9 patients actually tested positive on the cervical turn test pre-treatment.

† No inferential statistics reported

The included studies comprised five RCTs [26,76-79] and eight prospective, non-controlled, cohort studies [75,80-86], with samples sizes ranging from 12 to 168. One study [81] did not report on the gender distribution of recruited participants, however all but one of the remaining studies included more females, ranging from 52% to 88%.

Six studies [75-77,80-82], including two RCTs [76,77], used only spinal manipulation or mobilisation, or both, as the intervention. The remaining seven investigations [26,78,79,83-86], including three RCTs [26,78,79] utilised a multimodal approach consisting of several different interventions (e.g. spinal manipulation and mobilisation, soft tissue therapy, electrotherapy, and medications) and home exercise programs. However, none of the included studies used manual therapy in conjunction with vestibular rehabilitation.

Twelve studies, including all five RCTs, reported improvements in dizziness and associated symptoms (e.g. neck pain) following manual therapy intervention. The remaining study measured skull spatial offset repositioning ability, and found a significant improvement following soft tissue manipulation [75]. In addition to reduction in dizziness and associated symptoms, two RCTs [77,79] reported significant changes in vertebrobasilar artery blood flow velocity post-treatment, and a further two RCTs [26,76] found improvement in balance performance measured with posturography.

The methodological quality of the included studies was generally poor [75,80-85] to moderate [26,77-79,86]. However, one study [76] was found to be of good methodological quality. Not surprisingly, there was a trend towards more robust study designs (i.e. RCTs) and more recently published studies attaining higher quality scores. Overall, common methodological weaknesses included: lack of control group; failure to provide information allocation concealment and participant, provider, and assessor blinding; omitting performing appropriate statistical analysis; omitting reporting on patient compliance and drop-outs; and including long-term follow-up measurements. A tabulated overview of methodological quality scores is provided in Additional file 3.

Only three studies commented on adverse reactions. Two RCTs [26,76] reported no adverse reactions, and one prospective cohort study [86] found minor adverse reactions associated with the interventions in eight of nineteen participants.

## Discussion

In a previous review of the literature, Reid and Rivett [29] concluded there was limited (Level 3) evidence for manual therapy in the treatment of cervicogenic dizziness. The current systematic review has identified additional studies published since the previous review, including: four RCTs [76-79], three prospective cohort studies [75,85,86], and a long-term follow up [73] of the intervention group from the RCT published by Karlberg et al. [26].

The RCT by Reid et al. [76], which was deemed to be of good methodological quality, assessed the effectiveness of a specific type of spinal mobilisation known as sustained natural apophyseal glides (SNAGs). Reid et al. [76] found significant improvement in dizziness severity and frequency, lower scores on the Dizziness Handicap Inventory (DHI), and decreased neck pain in the treatment group at both six and twelve weeks post-treatment. In comparison the placebo group had significant changes only at the 12-week follow-up in three outcome measures (dizziness severity, DHI, and neck pain). The remaining four RCTs [26,77-79] were deemed to be of moderate methodological quality. The findings from the RCT by Karlberg et al. [26] (including the long-term follow-up by Malmstrom et al. [44] appear to corroborate the evidence provided by Reid et al. [76]. The RCTs by Kang, Wang and Ye [77], Fang [78], and Du et al. [79] all utilised spinal manipulation in the intervention group and reported improvements in clinical outcomes.

In addition to five RCTs the current systematic review identified eight prospective cohort studies, of which seven [80-86] reported improvements in dizziness following manual therapy. Although these were generally of poor methodological quality they also reported improvements in additional outcome measures, including: neck pain [86], reduction of pathological vestibulospinal activity [84], balance [86], and reduced vertebral displacement and vertebrobasilar artery blood flow velocity [85] The remaining cohort study [75] reported improvements in skull spatial offset repositioning ability post treatment. Collectively, these findings provide further rationale for the use of manual therapy in the treatment of cervicogenic dizziness. Overall, the evidence evaluated in the current systematic review suggests that there is moderate (Level 2) evidence in a favourable direction to support the use of manual therapy for cervicogenic dizziness.

Although positive clinical outcomes have been demonstrated, the underlying biological mechanism remains a controversial subject. It has been theorised that disturbances to the afferent input from cervical spine mechanoreceptors may lead to a sensory mismatch between vestibular and cervical input subsequently resulting in symptoms such as dizziness, unsteadiness, and visual disturbances [25]. There is an experimental body of evidence indicating that the biomechanical forces of spinal manipulation and mobilisation impacts primary afferent neurons in paraspinal tissues, which in turn leads to physiological consequences such as gating of nociception at the spinal cord and spinal reflex activity to alter muscle activity [87,88]. Thus it is believed that manual therapy serves to normalise disturbances to the afferent input from deep neck proprioceptors and their subsequent reflex arcs (e.g. cervico-collic, cervico-ocular, and tonic neck), which in turn restores the ability to utilise internal vestibular orienting information to resolve inaccurate information from the somatosensory and visual subsystems (i.e. reducing sensory mismatch) [89].

Alas, no experimental or observational studies reporting the effect of combining manual therapy and vestibular rehabilitation in the management of cervicogenic dizziness could be identified. Collins and Misukanis [36] and Schenk et al. [90] have published case studies in which they argue that manual therapy combined with vestibular rehabilitation may be superior in the treatment of cervicogenic dizziness. Notwithstanding the paucity of such investigations, consideration of vestibular dysfunction is paramount in patients with dizziness. Unilateral peripheral vestibular dysfunction can be characterised by complaints of dizziness, visual or gaze disturbances and balance impairment [34]. In a recent meta-analysis of vestibular rehabilitation for unilateral peripheral vestibular dysfunction is was concluded that vestibular rehabilitation is a safe and effective therapy [34].

The original vestibular rehabilitation protocols were developed by Cooksey [91] in 1946. These included: mental exercise, occupational therapy, physical exercise with the aim of restoring balance and joint position sense, and training of the eyes, to compensate for permanent vestibular dysfunction [91]. More recently, Hillier and Hollohan [34] stated vestibular rehabilitation may include: learning to coordinate eye and head movements, improving balance and walking skills, learning to bring on the symptoms to desensitize the vestibular system, patient education, coping strategies, and physical activity. There are four mechanisms of vestibular rehabilitation techniques that may contribute to its benefits, namely: (i) the compensatory response, (ii) adaptation, (iii) substitution, and (iv) postural control exercises. The compensatory responses are applied using motion to minimise the responsiveness to repetitive stimuli and to rebalance tonic activity within the vestibular nuclei. Adaptation for visual-vestibular interaction uses repetitive and provocative movements of the head and/or eyes to minimise error and restore vestibulo-ocular reflex gain. Substitution encourages the use of other sensory inputs to compensate for dysfunctional afferent systems. Postural control exercises and functional retraining are applied for movement behaviour and fitness.

The four mechanisms canvas a rationale for the inclusion of vestibular rehabilitation in the management of patients with cervicogenic dizziness. Stability and posture of the cervical spine is achieved by a combination of reflexes mediated by vestibular, visual and cervical sensory input [19]. The cerebellum plays an important role in integrating this sensory information [92]. It can be hypothesised that a well-integrated vestibulo-cerebellar system would be more capable of compensating for the altered cervical sensory input in cases of cervicogenic dizziness. Thus, one can argue that when normal cervical afferent input is compromised, vestibular rehabilitation may strengthen the vestibulo-cerebellar system to improve the ability to adapt to the situation. Further research to elucidate the effectiveness of manual therapy in conjunction with vestibular rehabilitation for cervicogenic dizziness is strongly recommended.

There are insufficient data to provide guidelines on dosage and frequency of manual therapy in general, and spinal manipulation in particular, especially in the context of management of cervicogenic dizziness. With this in mind, it is recommended that caution is taken when delivering any sensory stimulation in the form of manual therapy or vestibular rehabilitation, or both, to affect dysfunctions in the afferent system in patients with cervicogenic dizziness. Further research is necessary to determine appropriate treatment dosage, scheduling of interventions, and which manual therapy and vestibular rehabilitation techniques are most effective in managing patients with cervicogenic dizziness.

Methodological limitations of this systematic review included lack of blinding during the quality assessment and the quality and utility of the quality assessment tool itself. Meta-analysis of the finding was precluded by the lack of robust research methodologies and heterogeneity of outcome measures in the studies included in this systematic review.

## Conclusion

This systematic review has found that there is moderate (Level 2) evidence in a favourable direction to support the use of manual therapy (spinal mobilisation and/or manipulation) for cervicogenic dizziness. The evidence for combining manual therapy and vestibular rehabilitation in the management of cervicogenic dizziness remains inconclusive due to no observational and experimental studies investigating manual therapy in conjunction with vestibular rehabilitation. However, there is a reasonable rationale for utilising manual therapy in conjunction with vestibular rehabilitation for cervicogenic dizziness, and further research to elucidate the potential synergistic effects is strongly recommended.

## Competing interests

The authors declare that they have no competing interests.

## Authors' contributions

MBS, CVC and GB conceived of the study, participated in the design of the study, and helped to draft and edit the manuscript. RPL participated in the design and coordination of the study, helped to draft, edit and revise the manuscript. All authors read and approved the final manuscript.

## Supplementary Material

Additional file 1 **Maastricht-Amsterdam criteria list**. The Maastricht-Amsterdam criteria list is an instrument developed by van Tulder et al. [40] to assess methodological quality clinical trials. It consists of nineteen items that can be rated individually using one of three options: *yes, no*, or *don't know*. The overall methodological quality score is determined by adding up all of the 'yes' ratings, with a maximum score of nineteen. Click here for file

Additional file 2 **Excluded studies**. Alphabetic list of excluded studies, including the reasons for exclusion. Click here for file

Additional file 3 **Methodological quality assessment scores of included studies**. Methodological quality assessment scores of included studies. Click here for file
