# Supplementary material for: Manual therapy with and without vestibular rehabilitation for cervicogenic dizziness: a systematic review
Source: Chiropr Man Therap. 2011 Sep 18;19:21. doi: 10.1186/2045-709X-19-21 (PMC3182131; doi:10.1186/2045-709X-19-21)
Supplement: Additional file 1 — Maastricht-Amsterdam criteria list. The Maastricht-Amsterdam criteria list is an instrument developed by van Tulder et al. [40] to assess methodological quality clinical trials. It consists of nineteen items that can be rated individually using one of three options: yes, no, or don't know. The overall methodological quality score is determined by adding up all of the 'yes' ratings, with a maximum score of nineteen. [file 2045-709X-19-21-S1.DOC]

**Additional file 1. Maastricht-Amsterdam criteria list**

*Patient Selection*

A: Were the eligibility criteria specified

B1: Was a method of randomization performed

B2: Was the treatment allocation concealed

C: Were the groups similar at baseline regarding the most important prognostic indicators

*Intervention*

D: Were the index and control interventions explicitly described

E: Was the care provider blinded to the intervention

F: Were co-interventions avoided or comparable

G: Was the compliance acceptable in all groups

H: Was the patient blinded to intervention

*Outcome Measurement*

I: Was the outcome assessor blinded to the intervention

J: Were the outcome measures relevant

K: Were adverse effects described

L: Was the withdrawal/drop-out rate described and acceptable

M1: Was a short term follow-up measurement performed

M2: Was a long term follow-up measurement performed

N: Was the timing of the outcome measurement in both groups comparable

*Statistics*

O: Was the sample size for each group described

P: Did the analysis include an intention-to-treat analysis

Q: Were point estimates and measures of variability presented for the primary outcome measures
