# Supplementary material for: Manual therapy with and without vestibular rehabilitation for cervicogenic dizziness: a systematic review
Source: Chiropr Man Therap. 2011 Sep 18;19:21. doi: 10.1186/2045-709X-19-21 (PMC3182131; doi:10.1186/2045-709X-19-21)
Supplement: Additional file 2 — Excluded studies. Alphabetic list of excluded studies, including the reasons for exclusion. [file 2045-709X-19-21-S2.DOC]

**Additional file 2. Excluded studies.**

| **Study** | **Reason for exclusion** |
| --- | --- |
| Becker 1978 [44] | Study design: review |
| Biesinger 1987 [45] | Study design: review |
| Borg-Stein, Rauch and Krabak 2001 [46] | Study design: review |
| Bronfort et al. 2010 [47] | Study design: review |
| Collins and Misukanis 2005 [36] | Study design: case report |
| Eber 1994 [48] | Study design: review |
| El-Kahky et al. 2000 [49] | Study population: not cervicogenic dizziness  Intervention: no manual therapy |
| Falkenau 1976 [50] | Study design: case study |
| Garcia 2009 [51] | Study design: review |
| Grgic 2006 [52] | Study design: review |
| Grod and Diakow 2002 [53] | Study population: not cervicogenic dizziness  Intervention: not manual therapy |
| Hansson and Håkansson 2009 [54] | Study design: review |
| Hansson, Månsson and Håkansson 2005 [55] | Study design: retrospective  Study population: not cervicogenic dizziness |
| Hawk and Cambron 2009 [56] | Study population: not cervicogenic dizziness |
| Hawk et al. 2007 [57] | Study design: review |
| Heikkilä, Johansson and Wenngren 2000 [58] | Study design: case series |
| Jäger 2004 [59] | Study design: review |
| Jepsen 1963 [60] | Study design: review |
| Karlberg, Persson, Magnusson 1995 [61] | Study population: not cervicogenic dizziness |
| Maffei 1983 [62] | Study design: review |
| Persson, Karlberg and Magnusson 1996 [63] | Study population: not cervicogenic dizziness |
| Rapaccini and Pascucci 2003 [64] | Study population: not cervicogenic dizziness  Intervention: no manual therapy |
| Reid and Rivett 2005 [29] | Study design: systematic review |
| Rohmer and Collard 1974 [65] | Study design: review |
| Rong 2003 [66] | Study population: not cervicogenic dizziness |
| Scherer 1985 [67] | Study design: review |
| Seifert 1987 [68] | Study design: review |
| Takizawa et al. 1967 [69] | Study design: review |
| Teixeira and Prado 2009 [70] | Study population: not simply cervicogenic dizziness |
| Thomas 2009 [71] | Study design: review |
| Yardley et al. 1998 [72] | Intervention: no manual therapy  Study population: poor diagnostic criteria for cervicogenic dizziness |
