# Supplementary material for: Manual therapy with and without vestibular rehabilitation for cervicogenic dizziness: a systematic review
Source: Chiropr Man Therap. 2011 Sep 18;19:21. doi: 10.1186/2045-709X-19-21 (PMC3182131; doi:10.1186/2045-709X-19-21)
Supplement: Additional file 3 — Methodological quality assessment scores of included studies. Methodological quality assessment scores of included studies. [file 2045-709X-19-21-S3.DOC]

**Additional file 3. Methodological q**uality assessment

| **Study** | **A** | **B1** | **B2** | **C** | **D** | **E** | **F** | **G** | **H** | **I** | **J** | **K** | **L** | **M1** | **M2** | **N** | **O** | **P** | **Q** | **Total** |
| --- | --- | --- | --- | --- | --- | --- | --- | --- | --- | --- | --- | --- | --- | --- | --- | --- | --- | --- | --- | --- |
| Konrad and Gerencser 1990 [80] | 0 | 0 | 0 | 0 | 0 | 0 | 1 | 0 | 0 | 0 | 1 | 0 | 0 | 1 | 0 | 1 | 0 | 0 | 0 | 4 |
| Mahlstedt, Westhofen and König 1992 [81] | 0 | 0 | 0 | 0 | 0 | 0 | 1 | 0 | 0 | 0 | 1 | 0 | 0 | 1 | 0 | 1 | 0 | 0 | 0 | 4 |
| Uhlemann et al. 1993 [82] | 0 | 0 | 0 | 1 | 0 | 0 | 1 | 0 | 0 | 0 | 1 | 0 | 0 | 1 | 0 | 1 | 1 | 0 | 0 | 6 |
| Karlberg et al. 1996 [26] &  Malmström et al. 2007 [73] | 1 | 1 | 0 | 1 | 1 | 0 | 0 | 0 | 0 | 0 | 1 | 1 | 1 | 1 | 1 | 1 | 1 | 0 | 1 | 12 |
| Bracher et al. 2000 [83] | 1 | 0 | 0 | 0 | 0 | 0 | 0 | 0 | 0 | 0 | 1 | 0 | 1 | 1 | 0 | 0 | 1 | 0 | 0 | 5 |
| Hülse and Hölzl 2000 [84] | 0 | 0 | 0 | 0 | 1 | 0 | 0 | 0 | 0 | 0 | 1 | 0 | 0 | 1 | 0 | 1 | 1 | 0 | 1 | 6 |
| Chen and Zhan 2003 [85] | 1 | 0 | 0 | 0 | 1 | 0 | 0 | 1 | 0 | 0 | 1 | 1 | 0 | 1 | 0 | 0 | 1 | 0 | 1 | 8 |
| Kang, Wang and Ye 2008 [77] | 1 | 1 | 0 | 1 | 1 | 0 | 0 | 1 | 0 | 0 | 1 | 0 | 0 | 1 | 1 | 1 | 1 | 0 | 1 | 11 |
| Reid et al. 2008 [76] | 1 | 1 | 1 | 1 | 1 | 0 | 1 | 1 | 1 | 1 | 1 | 1 | 1 | 1 | 1 | 1 | 1 | 1 | 1 | 18 |
| Wu et al. 2008 [74] | 1 | 0 | 0 | 0 | 1 | 0 | 1 | 0 | 0 | 0 | 1 | 0 | 0 | 1 | 0 | 1 | 1 | 0 | 1 | 8 |
| Strunk and Hawk 2009 [86] | 1 | 0 | 0 | 1 | 1 | 0 | 0 | 1 | 0 | 1 | 1 | 1 | 1 | 1 | 0 | 1 | 1 | 0 | 1 | 12 |
| Du et al. 2010 [79] | 1 | 1 | 0 | 1 | 1 | 0 | 1 | 1 | 0 | 0 | 1 | 0 | 0 | 1 | 1 | 1 | 1 | 0 | 1 | 12 |
| Fang 2010 [78] | 1 | 1 | 0 | 1 | 1 | 0 | 1 | 1 | 0 | 0 | 1 | 0 | 0 | 1 | 0 | 0 | 1 | 0 | 1 | 10 |
